# Readability Comparison of AI-Generated Versus UpToDate Educational Content on Stroke Management: A Cross-Sectional Study

**DOI:** 10.7759/cureus.98901

**Published:** 2025-12-10

**Authors:** Saow Renn Ding, Mohammed Ahmed, Tazeen Malik, Rashmitha Somagani, Faizaan Farukh Vohra

**Affiliations:** 1 Medicine, St. George’s University School of Medicine, St. George, GRD; 2 Stroke/Neurology, Salford Royal Hospital, Greater Manchester, GBR; 3 Neurology, Lake Erie College of Osteopathic Medicine, Erie, USA; 4 Neurology, Employees' State Insurance Corporation (ESIC) Medical College and Hospital, Hyderabad, IND; 5 Emergency, Sterling Hospital, Vadodara, IND

**Keywords:** artificial intelligence, chatgpt, medical education, readability score, stroke, uptodate

## Abstract

Introduction

Stroke is a major cause of global morbidity and mortality. Readability of educational material is critical for rapid clinical decision-making among healthcare professionals. UpToDate (UpToDate, Inc., Waltham, MA) is a widely used, peer-reviewed point-of-care clinical resource, while ChatGPT (OpenAI, San Francisco, CA) is an emerging AI-based educational support tool. However, a formal comparison of their linguistic accessibility has not been performed.

Objective

To compare the readability and linguistic complexity of educational material on stroke generated by ChatGPT (GPT-4o) versus content retrieved from UpToDate, using validated readability metrics.

Design, setting, and participants

This cross-sectional study was conducted between May 27 and June 4, 2025. ChatGPT (GPT-4o, accessed May 27, 2025) was prompted to generate educational content on stroke. A corresponding section from UpToDate (accessed May 27, 2025) was extracted. Only prose content was analyzed. Readability parameters assessed included total word count, sentence count, word/sentence ratio (average words per sentence), Flesch Reading Ease (FRE), Flesch-Kincaid Grade Level (FKGL), Simple Measure of Gobbledygook (SMOG) Index, difficult word count, and difficult word percentage. Data were analyzed using IBM SPSS v25 (IBM Corp., Armonk, NY) and R v4.3.2 (R Foundation for Statistical Computing, Vienna, Austria). The Mann-Whitney U test was used. P < 0.05 was considered statistically significant.

Results

UpToDate content was substantially longer (median = 2772 vs. 304 words; p = 0.008) and used more sentences (median = 134 vs. 23; p = 0.032) and difficult words (median = 857 vs. 88; p = 0.008) compared to ChatGPT. The word/sentence ratio (average words per sentence) was also higher (21.7 vs. 13.2; p = 0.008). However, no statistically significant differences were observed for FRE (p = 1.000), FKGL (p = 0.222), SMOG Index (p = 0.151), or difficult word percentage (p = 0.690).

Conclusions

ChatGPT produces shorter and more concise educational content on stroke while maintaining comparable readability to UpToDate. The lower linguistic density may enhance rapid orientation for trainees; however, the reduced depth indicates ChatGPT should supplement, not replace, established peer-reviewed resources. Future research should explore multiple medical topics, additional AI models, and assess the clinical applicability and accuracy of AI-generated content.

## Introduction

Stroke is currently the world’s third-leading cause of death and disability combined, accounting for an estimated 7.3 million deaths and 160 million disability-adjusted life-years in 2021; global prevalence reached ~94 million people, and incident strokes rose by 70% compared with 1990 [1]. The 2024 American Heart Association/American Stroke Association (AHA/ASA) guidelines on primary stroke prevention stress that aggressive control of vascular risk factors and lifestyle modification could prevent up to 80% of first strokes when implemented across the lifespan [2]. In urgent clinical scenarios, the speed at which clinicians can understand and apply evidence-based recommendations is crucial, making the readability of educational material a key factor in effective decision-making.

UpToDate is an extensively subscribed, peer-reviewed point-of-care (POC) resource used by more than three million clinicians worldwide. Its institutional use has been associated with shorter hospital stays, lower mortality risk, and improved quality metrics [3]. However, even experienced care providers may experience information overload in fast-paced environments such as acute stroke, where concise and readable content can help accelerate assimilation of recommendations.

Large language model (LLM) tools have emerged as alternatives for just-in-time learning. A recent American Medical Association (AMA) survey reported that two-thirds of US physicians used some form of healthcare AI in 2024, marking a 78% increase from the previous year. OpenAI (San Francisco, CA) tools, including GPT-4 Turbo and GPT-4o, have been used for tasks such as clinical assistance and content drafting, with ChatGPT being the most widely adopted for educational purposes [4]. However, existing research shows mixed results regarding the readability of AI-generated medical content. Some studies report Flesch Reading Ease Scores (FRES) in the low 30s, equivalent to an undergraduate reading level, when typical prompting is used [5], while others note that even when instructed to “write at a fourth-grade level,” ChatGPT often produces content that remains complex and less comprehensive than patient-oriented materials [6]. Qualitative evaluations of AI-generated discharge summaries identify stylistic inconsistencies and omission of clinical nuances [7], and citation hallucination rates ranging from 10% to almost 50% have also been reported in scientific contexts [8].

To date, no study has benchmarked the readability of clinician-level educational content on a high-stakes topic such as stroke between an LLM and a peer-reviewed POC reference. This study aims to compare the readability and linguistic complexity of ChatGPT-generated educational content with that retrieved from UpToDate (UpToDate, Inc., Waltham, MA), evaluating whether AI-generated material can match or surpass the linguistic accessibility of expert-curated resources.

## Materials and methods

This cross-sectional original research study was conducted over one week, from May 27 to June 4, 2025. As the study did not involve human participants, identifiable data, or any medical interventions, approval from an Institutional Ethics Committee was not required [9,10].

The topic selected for analysis was stroke, a clinically significant and commonly encountered neurological condition. The primary objective was to compare the readability of educational material generated by an AI language model with that from an established evidence-based clinical resource.

Educational content aimed at medical professionals was generated using ChatGPT (GPT-4o, accessed on May 27, 2025), developed by OpenAI. The following standardized prompt was used: “Write an educational guide for medical professionals on stroke, including definition, clinical features, diagnosis, and treatment options.” Default model settings, including temperature, were used to reflect typical usage conditions. The generated text was copied into a Microsoft Word document (Microsoft Corporation, Redmond, WA) for analysis.

For comparison, stroke-related clinical content was retrieved from UpToDate (version accessed May 27, 2025). Only the main body of the disease summary was included. Supplementary elements such as tables, references, figures, and bullet-pointed material were excluded to ensure consistency in prose-based analysis.

Readability was assessed using the Flesch Reading Ease (FRE) score, Flesch-Kincaid Grade Level (FKGL), and Simple Measure of Gobbledygook (SMOG) Index, calculated via an online Flesch-Kincaid calculator as per references [11,12]. Quantitative parameters evaluated included total word count, sentence count, average words per sentence (word/sentence ratio), FRE score, FKGL, SMOG index, difficult word count, and difficult word percentage.

All data were compiled using Microsoft Excel (Microsoft Corporation) and analyzed using R software (version 4.3.2; R Foundation for Statistical Computing, Vienna, Austria). Descriptive statistics were calculated (median and interquartile range). The Mann-Whitney U test was applied due to non-normal distribution and small sample size. A p-value < 0.05 was considered statistically significant.

As only one medical topic and a single content output from each source were evaluated, reproducibility may be affected by future updates to AI models or clinical guidelines. This has been acknowledged as a study limitation.

## Results

The responses by ChatGPT and UpToDate were evaluated based on readability parameters, including word count, sentence count, word/sentence ratio, FRE, FKGL, SMOG Index, difficult word count, and difficult word percentage.

Table 1 presents a comparison of readability characteristics between ChatGPT and UpToDate. The analysis was performed using IBM SPSS (version 25; IBM Corp., Armonk, NY) and R (version 4.3.2). Mann-Whitney’s U test was used to compare the distribution of responses generated by UpToDate and ChatGPT. Based on the p-values obtained in Table 1, there is a statistically significant difference between the median word count, sentence count, word/sentence count, and difficult word count generated by the two AI tools.

**Table 1:** Comparison of readability characteristics between ChatGPT and UpToDate. ^+ ^Mann-Whitney’s U test. * P-values <0.05 are considered statistically significant. FRE: Flesch Reading Ease; FKGL: Flesch-Kincaid Grade Level; SMOG: Simple Measure of Gobbledygook.

Parameters	Median (IQR)		U statistic	P-value^+^
	UpToDate	ChatGPT		
Word count	2772.0 (1003.5 – 6776.0)	304.0 (175.0 – 600.5)	0	0.008*
Sentence count	134.0 (46.0 – 314.0)	23.0 (10.5 – 58.0)	2	0.032*
Word/sentence count	21.7 (20.2 – 23.2)	13.2 (10.7 – 17.8)	0	0.008*
FRE	20.6 (15.7 – 28.7)	20.4 (14.6 – 27.3)	12	1.000
FKGL	16.2 (14.6 – 16.4)	13.3 (12.3 – 15.6)	6.5	0.222
SMOG Index	14.2 (13.1 – 14.4)	10.9 (10.4 – 13.6)	5	0.151
Difficult word count	857.0 (280.0 – 1575.5)	88.0 (47.5 – 190.5)	0	0.008*
Difficult word percentage	27.5 (23.4 – 29.6)	30.1 (22.2 – 31.6)	10	0.690

The results demonstrate statistically significant differences in several metrics. UpToDate responses had a significantly higher median word count compared to ChatGPT (P = 0.008). Similarly, the sentence count was higher for UpToDate versus ChatGPT (P = 0.032). The word-to-sentence ratio was also significantly greater for UpToDate compared to ChatGPT (P = 0.008). Additionally, the number of difficult words used was markedly higher in UpToDate (P = 0.008).

However, there were no statistically significant differences between the two sources in terms of readability scores. The FRE scores were nearly identical (P = 1.000). Similarly, no significant differences were found in the FKGL (P = 0.222), SMOG Index (P = 0.151), or the percentage of difficult words (P = 0.690).

**Figure 1:**
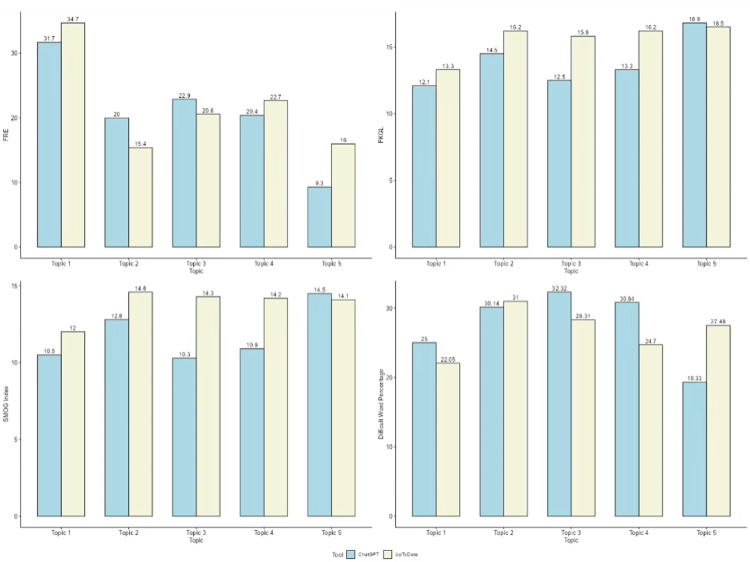
Graphical representation of comparison between FRE, FKGL, SMOG Index, and difficult word percentage for the patient education guides generated by UpToDate and ChatGPT. FRE: Flesch Reading Ease; FKGL: Flesch-Kincaid Grade Level; SMOG: Simple Measure of Gobbledygook.

Figure 1 shows a graphical representation of the comparison between FRE, FKGL, SMOG Index, and difficult word percentage for the patient education guide generated by UpToDate and ChatGPT. The findings indicate that ChatGPT generally produces content that is easier to read compared to UpToDate. In terms of FRE, ChatGPT typically scores higher or similarly across all topics, suggesting better readability, although Topic 5 is an exception, where both are difficult to read, but UpToDate scores slightly better (16 vs. 9.3 for ChatGPT). For the FKGL, ChatGPT consistently scores lower, meaning it uses simpler language that requires a lower educational level to understand. Notable differences appear in Topic 3 (ChatGPT: 12.5 vs. UpToDate: 15.8) and Topic 4 (ChatGPT: 13.3 vs. UpToDate: 16.2), indicating significantly easier comprehension with ChatGPT responses. The SMOG Index also favors ChatGPT, with slightly lower scores in most topics, suggesting simpler vocabulary and sentence structures, except in Topic 5, where ChatGPT scores marginally higher (14.5 vs. 14.1). Lastly, difficult word percentage is consistently lower in ChatGPT responses, with the most significant gap observed in Topic 5 (ChatGPT: 19.33% vs. UpToDate: 27.48%). Overall, while UpToDate tends to produce longer and more complex content, ChatGPT offers more accessible and easier-to-understand responses.

## Discussion

Overall, while UpToDate content was more extensive and complex, both platforms provided educational material of comparable readability. AI tools like ChatGPT are playing an increasingly important role in medical education by offering concise and accessible summaries that aid in rapid information retrieval. These tools support just-in-time learning by allowing clinicians to access key clinical concepts without the need to consult lengthy textbooks or databases. Especially for early-career professionals and trainees, AI-generated content can serve as a helpful starting point for understanding complex topics, complementing established resources like UpToDate and PubMed [13]. By streamlining the learning process and enhancing accessibility, AI tools contribute to more efficient and flexible medical education [14].

Readability scores are essential tools used to assess how easily a piece of text can be understood by readers. Commonly used metrics include the FRE, FKGL, and the SMOG Index. Higher FRE scores indicate easier readability, while lower FKGL and SMOG scores suggest content that is more accessible to a broader audience. In medical education, improved readability is crucial as it enables clinicians to quickly comprehend and apply information in time-sensitive clinical settings [15]. In this study, ChatGPT demonstrated slightly higher readability than UpToDate based on lower FKGL and SMOG Index scores, although the differences were not statistically significant. This suggests that ChatGPT's content may be somewhat easier to comprehend, potentially benefiting trainees and busy healthcare professionals seeking rapid understanding.

The findings of this study align with several previous studies examining AI-generated content in medical education. For example, one study showed that when prompting AI to convert patient educational material to an easier grade level, AI could significantly improve the readability of input material [16]. However, without prompting, the baseline reading level of ChatGPT-generated information is often much higher than is recommended for patient educational materials [17]. Another study found comparable trends in readability and structural clarity across different clinical scenarios [18]. Mondal et al. similarly found that LLMs can produce plain language summaries with significantly better readability than human-written text, while maintaining comparable overall quality, highlighting the growing role of AI tools in improving linguistic accessibility for clinicians and researchers [19]. Similarly, Sarangi et al. reported that ChatGPT was able to simplify complex radiological reports by removing technical jargon while preserving essential diagnostic information, further supporting the model’s capacity to enhance accessibility of specialized medical content for clinicians and patients [20]. These consistent patterns suggest that AI tools like ChatGPT perform reliably across various medical topics, reinforcing their potential as valuable supplements to conventional educational platforms in supporting medical professionals.

Another study shows that the challenges with ChatGPT in the education sector are well-recognized due to the lack of well-developed guidelines and ethical codes around generative AI [21]. Additionally, differences in study design, evaluation criteria, and reviewer backgrounds may contribute to inconsistent findings. These variations underscore the need for standardized methods to assess the quality, accuracy, and applicability of AI-generated content in medical education.

Limitations

This study is limited by the analysis of only one medical topic (stroke) and a single output from each tool, which restricts generalizability. AI-generated content may vary between sessions due to model updates or parameter changes, affecting reproducibility. The exclusion of tables, figures, and reference-linked material may influence readability outcomes. Additionally, only linguistic accessibility was assessed; clinical accuracy, contextual depth, and alignment with guidelines were not evaluated.

## Conclusions

In this cross-sectional study comparing ChatGPT-generated educational content with that from UpToDate, ChatGPT produced markedly shorter and more concise material while maintaining comparable readability levels. This suggests that AI-generated text may facilitate faster initial orientation for trainees and early-career professionals, although its reduced linguistic depth may limit standalone educational or clinical utility. The findings highlight ChatGPT’s potential as a supplementary learning tool rather than a replacement for expert-curated, peer-reviewed clinical resources.

Future research should include multiple medical topics, incorporate additional AI models, assess reproducibility across different prompt iterations, and evaluate whether improved readability translates to enhanced clinician comprehension, decision-making accuracy, and patient outcomes. Studies should further consider ethical oversight, content transparency, reliability, and integration of readability optimization with validated medical knowledge to ensure both educational clarity and clinical safety.
